# Editorial: Impact of sex and gender on neurocognitive aging and behavior

**DOI:** 10.3389/fnagi.2024.1524188

**Published:** 2024-12-09

**Authors:** Fereshteh Farajdokht, Craig Myrum, Clive R. Bramham, Akash Gautam

**Affiliations:** ^1^Neuroscience Research Center, Tabriz University of Medical Sciences, Tabriz, Iran; ^2^Department of Biology, Loyola University Maryland, Baltimore, MD, United States; ^3^Department of Biomedicine, University of Bergen, Bergen, Norway; ^4^Centre for Neural and Cognitive Sciences, University of Hyderabad, Hyderabad, India

**Keywords:** gender, sex, aging, cognition, brain function, behavior

Aging is often accompanied by declines in cognitive function and behavior. These changes include subtle deficits in memory to the devastating effects of Alzheimer's disease (AD) and substantial inter-individual variability in gait function among older adults. Not only do such differences exist between individuals, but many age-related health outcomes, vulnerabilities, and therapeutic responses also vary by sex and gender. This Research Topic highlights some of these differences to advance our understanding of the complex interplay between sex, gender, hormones, and other factors in neurocognitive aging and behavior.

The cross-sectional study by Ma et al. was conducted to examine the prevalence and risk factors of subjective cognitive decline (SCD) among 1,120 older adults aged 60+. The study demonstrated a high prevalence of SCD in this population, with 37.9%. Numerous risk factors for SCD were identified, such as advanced age, obesity, long-term smoking, coronary heart disease, visual impairment, vegetarian diet, average sleep duration <6 h per night, unmarried status, and residing in a rural region. Gender-based analysis revealed statistically significant differences in SCD prevalence across marital status, dietary habits, sleep duration, smoking, drinking, and hypertension. Given the higher prevalence of SCD in rural populations compared to urban ones, it suggests that socio-cultural influences, such as lower educational attainment and reduced opportunities for cognitive engagement, may contribute to diminished cognitive reserve and increased vulnerability to cognitive deterioration. These results underscore the substantial cognitive health challenges that the elderly encounter and offer valuable insights for the development of public health strategies and targeted interventions. The study underscores the significance of early screening and prevention programs, as well as the potential advantages of fostering physical activity and social engagement in the preservation of cognitive health in geriatric populations.

Sex differences were also observed in the study by Wang et al. where men displayed a greater degree of age-related cerebellar atrophy than women. The authors utilized convolutional neural networks to segment the cerebellum into 28 subregions and measured subregional volumes and shape analysis to measure cerebellar thickness. In addition to significant sex differences, the study supports earlier reports of region-specific changes in cerebellar volume, as well as region-specific rates of decline.

Despite growing recognition of substantial sex differences in aging, including those described above, studies specifically focusing on the effects of aging in women are lacking. This Research Topic also includes studies that aim to address this gap in knowledge. The narrative review by Warren looks into the complex relationships between neuroinflammation, AD, and gender-specific dietary habits, emphasizing the substantial gender disparity in the prevalence and severity of AD. The research demonstrates that males have a preference for red and processed meats, while women tend to favor healthier options such as vegetables and whole grains. Additionally, the study identifies distinct dietary preferences and eating patterns between the genders. Additionally, women demonstrate increased morning appetite and more frequent snacking. The review suggests that these gender-specific dietary practices may contribute to neuroinflammatory states that influence the pathogenesis of AD. The study offers valuable insights into the potential impact of diet on neuroinflammation and risk of AD by investigating a variety of factors that influence dietary choices, including biological, psychological, sociocultural, and socioeconomic influences. The significance of gender differences in nutritional approaches to cognitive health and AD prevention is emphasized by this research, which has the potential to inform targeted interventions and care practices in the field of neurodegenerative diseases.

The cross-sectional study by Wu et al. examined the correlation between cognitive function and physical activity (PA) patterns in elderly women, utilizing data from the National Health and Nutrition Examination Survey (NHANES) 2011–2014. The study encompassed 1,507 female participants aged 60 and older, and it identified various physical activity patterns, including Inactive, Weekend Warrior (WW), and Regular Exercise (RE). The research uncovered substantial disparities in terms of health status indicators, lifestyle factors, and sociodemographic characteristics among participants with varying PA patterns. The research uncovered associations between cognitive function and PA patterns, underscoring the potential protective effects of regular exercise and active social participation against cognitive decline. These results highlight the necessity of targeted interventions and public health strategies to encourage active lifestyles in elderly women and emphasize the significance of physical activity in maintaining cognitive health. The research provides valuable insights to the expanding body of evidence that establishes a correlation between physical activity and cognitive health in senior adults, with a particular emphasis on elderly women.

The study by Chen et al. found that older women have decreased stability during gait initiation compared to younger adults, especially in complex environments. Older adults tend to have longer anticipatory postural adjustments and lower mediolateral stability to ensure safety when transitioning from double-leg to single-leg support. The length of the first step is a significant predictor of postural stability issues in older adults. Cognitive demands significantly influence the stability of gait initiation tasks in older adults, as they respond by prolonging lateral displacement time. This compensatory mechanism can lead to reduced step length and velocity, increased step width and foot inclination angle, and increased fear of falling. When cognitive tasks are time-pressured, older adults may prioritize forward propulsion over stability, increasing fall risk. The study highlights the importance of considering gender differences in fall risk management and tailoring interventions to address the unique challenges faced by older women.

Finally, in the paper by Nerattini et al. the authors note that while neuroprotective effects of estrogen are well-documented in women, the effect of menopausal hormone therapy on AD risk reduction is less clear. To that end, they carried out a large meta-analysis of randomized controlled trials and observational studies. Their stratified analysis estimated a 32% reduced risk of dementia with midlife estrogen therapy alone, while estrogen-plus-progesterone therapy was less effective, as was late-life hormone therapy.

Together this Research Topic of studies serves to illustrate the wide impacts of sex and gender on neurocognitive aging and behavior and underscores the knowledge gaps and challenges in this still-emerging field.

